# Renewable Poly(Lactic Acid)Lignocellulose Biocomposites for the Enhancement of the Water Retention Capacity of the Soil

**DOI:** 10.3390/polym15102243

**Published:** 2023-05-09

**Authors:** Dalila Rubicela Cruz Fabian, Silvie Durpekova, Miroslava Dusankova, Jaroslav Cisar, Petra Drohsler, Ondrej Elich, Marketa Borkova, Jarmila Cechmankova, Vladimir Sedlarik

**Affiliations:** 1 Centre of Polymer Systems, University Institute, Tomas Bata University in Zlin, Tr. T. Bati 5678, 760 01 Zlin, Czech Republic; cruz_fabian@utb.cz (D.R.C.F.);; 2 Dairy Research Institute, Ke Dvoru 12a, 160 00 Prague, Czech Republic; 3 Research Institute for Soil and Water Conservation, Zabovreska 250, 15627 Prague, Czech Republic

**Keywords:** lignocellulose waste, wheat straw, sawdust, poly(lactic acid), water retention, agriculture

## Abstract

This manuscript details the preparation and characterization of a renewable biocomposite material intended as a soil conditioner based on low-molecular-weight poly(lactic acid) (PLA) and residual biomass (wheat straw and wood sawdust). The swelling properties and biodegradability of the PLA-lignocellulose composite under environmental conditions were evaluated as indicators of its potential for applications in soil. Its mechanical and structural properties were characterized by differential scanning calorimetry (DSC), thermogravimetric analysis (TGA), Fourier-transform infrared spectroscopy (FTIR), and scanning electron microscopy (SEM). Results showed that the incorporation of lignocellulose waste material into PLA increased the swelling ratio of the biocomposite by up to 300%. The application of the biocomposite of 2 wt% in soil enhanced its capacity for water retention by 10%. In addition, the cross-linked structure of the material proved to be capable of swelling and deswelling repeatedly, indicating its good reusability. Incorporating lignocellulose waste in the PLA enhanced its stability in the soil environment. After 50 days of the experiment, almost 50% of the sample had degraded in the soil.

## 1. Introduction

Growing water scarcity has emerged as the most limiting factor for crop production in agriculture, particularly in arid and semi-arid regions, and the world is searching for agricultural practices that promote water use efficiency [1]. Water-absorbing polymers are one way to enhance the water use efficiency of the soil and contribute towards conditions that are beneficial to plant growth [2]. This material is capable of absorbing large amounts of water without dissolving and desorbing the absorbed water into the soil when exposed to drought. As a soil conditioner, it improves the water retention ability of the soil and increases nutrient availability for plants. Water-absorbing materials can also act as an agent for slow-release fertilizers to minimize nutrient losses by leaching and thus decrease pollution with respect to the environment [1,2,3]. In addition, the application of such materials to soil improves soil structure, permeability, water infiltration and evaporation rates while reducing water run-off and erosion, which inversely improve crop productivity and feasible crop yields [2,4]. However, most of these materials available on the market are synthesized from petroleum-based polymers, and their high costs and hampered biodegradability have restricted applications at the agricultural scale [5]. As a direct result of the negative impact of synthetic polymers on the environment, researchers have focused on the development of a novel sustainable bipolymer based on renewable resources [6,7]. Using nature-sourced biopolymers such as proteins, polysaccharides or their combination has advantages over synthetic forms due to their low cost, inherent hydrophilicity, biodegradability, and environmental safety [4,8]. However, some bio-based hydrogels have shown poor mechanical properties, which greatly limit their application prospects [9]. The use of lignocellulosic biomass and/or its constituent components for the preparation of water-absorbing materials (hydrogels) has shown great potential in this regard, as it can significantly improve mechanical properties while maintaining their biocompatibility [10]. There are a number of studies dealing with the preparation of biopolymer composites based on lignocellulosic biomass for various applications. For example, hydrogels derived from lignocellulosic biomass have attracted growing interest in the agriculture industry due to their low price, good swelling properties, biocompatibility and biodegradability [10].

Agricultural residues such as wheat straw (WS) and wood waste sourced from the wood industry, e.g., sawdust (SD), constitute highly abundant natural lignocellulose fillers that are especially applicable to the preparation of numerous environmentally friendly, renewable materials [5,11]. WS and SD fillers possess a natural structure of cellulose fibers in the form of an amorphous matrix of hemicellulose and lignin, which contains a number of strongly hydrophilic hydroxyl groups [12]. These groups are capable of triggering a chain of chemical reactions (e.g., copolymerization, esterification and etherification) and increasing the extent of water absorption or the desorption of composites when exposed to changes in the relative humidity of the environment [13]. Moreover, residual lignin within the polymer matrix might slow down biodegradation [5]. Therefore, they can be successfully employed in the development of numerous renewable and environmentally friendly water-absorbing materials with desirable swelling and mechanical properties [5,6,12,14,15]. For instance, Heise et al. [5] fabricated wheat-straw-based hydrogels, starting from a carboxymethylated lignocellulosic matrix for agricultural use as a soil conditioner. Zhang et al. [16] prepared novel hydrogel slow-release nitrogen fertilizers based on sawdust cellulose. Li et al. [17] developed a novel controlled-release fertilizer based on wheat straw cellulose hydrogel. However, no raw lignocellulosic materials have been used for the preparation of the water-absorbing material intended as a soil conditioner for agricultural applications.

This work describes the preparation of a natural biopolymeric soil conditioner fabricated from residual biomass sourced from the agriculture and wood industry (WS and SD) and a low-molecular-weight poly(lactic acid) (PLA) by a simple process that does not require any pre-treatments involving the extraction of raw cellulosic materials, permitting the utilization of raw lignocellulose waste. Thanks to the inexpensive production and facile degradation of the composite, it can be used in agriculture on a large scale to increase water retention in soil and support crop growth. In addition, the use of lignocellulose waste products from agriculture and the wood industry reduces both the quantity of such industrial waste and the negative environmental effects associated with extracting cellulosic materials. 

PLA is a renewable, biodegradable aliphatic polyester that exhibits favorable properties, such as good processability and mechanical performance, non-toxicity and biodegradability [18]. These excellent properties mark it as an environmentally friendly material suitable for various industrial applications, including agriculture, as an alternative to petroleum-based polymers [19]. Blending PLA with lignocellulose materials could improve biodegradability and increase the capacity for the water absorption of the PLA/lignocellulose composite and subsequently enhance water retention in soil [6]. Polycarboxylic acid was found to effectively cross-link lignocellulosic materials without the need for an initiator or organic solvent, also attracting interest as a component in the preparation of absorbent materials due to its inexpensive, natural origin and non-toxicity [5]. CA was selected as a cross-linking agent to form a cross-linking structure via the esterification reaction. The esterification of lignocelluloses refers to the reaction between the hydroxyl groups on the molecular chains and carboxylic acids. CA can react with cellulose, hemicellulose and lignin under mild conditions to form a cross-linking structure [20]. This mechanism is based on an anhydride intermediate formation [21].

In this work, the physicochemical properties of PLA-lignocellulose biocomposites were studied to evaluate their potential for applications in the soil as a soil conditioner in order to increase water retention.

## 2. Materials and Methods

### 2.1. Materials

A low-molecular-weight PLA (Mw~7 700) was synthesized from L-Lactic acid (L(+)-LA 80% (C_3_H_6_O_3_, Lach-Ner, Neratovice, Czech Republic) by a polycondensation reaction described below. Tin (II) 2-ethyl hexanoate (Sn(Oct)_2_, Sigma Aldrich, Milano, Italy) constituted the catalyst during synthesis, and acetone (C_3_H_6_O, VWR International, Stribrna Skalice, Czech Republic) was employed as the solvent of the resultant PLA. Methanol (CH_4_O, VWR International, Stribrna Skalice, Czech Republic) was used to clean the fabricated PLA, while citric acid (CA) in an anhydrous form (Lach-Ner, Neratovice, Czech Republic) was applied as a natural, non-toxic cross-linker of carboxylic groups in the given lignocellulosic materials supplementing the PLA. Chopped WS and SD were sourced as lignocellulosic waste products of agricultural activities and the wood industry in the territory of the Czech Republic.

### 2.2. Synthesis of PLA

The low-molecular-weight PLA was synthesized from lactic acid (LA) in accordance with a procedure by Kucharczyk et al. [22]. This commenced with LA at an amount of 400 mL that was dosed into a three-neck round-bottom flask equipped with a Teflon stirrer that was placed in an oil bath and heated at 160 °C for 3 h to remove water; for this purpose, a heating magnetic stirrer (Phoenix RSM-02 HP) was connected to laboratory apparatus for distillation under reduced pressure (20 kPa). The reaction vessel was disconnected from the vacuum pump, and 0.5 wt% of the Tin(II) 2-ethylhexanoate catalyst (related to the initial mass of the reactants) was dropwise added into it under continuous stirring at 300 rpm. The flask was then reconnected to the vacuum source at 100 Pa, and the reaction continued for 48 h at 160 °C. The resulting product was cooled to room temperature and dissolved in acetone. The polymer solution subsequently underwent precipitation in a mixture of chilled methanol and distilled water at a ratio of 1:1 (*v*/*v*) and was then filtrated by centrifugation at 13,000 rpm; this process of dissolution and precipitation was repeated twice more. The final product was washed with distilled water and dried at 45 °C for 48 h. The resultant pure PLA had a molecular weight of 7700 g/mol, as characterized by GPC analysis (Agilent PL-GCP 220 chromatographic system).

### 2.3. Preparation of the PLA/Lignocellulose Composite Material

The low-molecular-weight PLA in powder form was dissolved in acetone under continuous stirring at 45 °C for 30 min to obtain a PLA solution of 10 wt% (Solution 1). The cross-linking agent (CA, 10 wt% of polymer weight) was dissolved in distilled water under continuous stirring at 70 °C for 15 min (Solution 2). The WS and SD were ground in an IKA A11 B mixer and sieved to a particle size of ≤300 µm and the particle size distribution was determined on a test sieve shaker (HAVER EML 200 digital plus T). The relevant amount of WS or SD (15, 35, and 60 wt%) was then added into Solution 2 at a mass ratio of 1:8 to enhance the dissolution of components, followed by thorough mixing for 30 min. This solution was heated at 100 °C, as such pre-treatment aids the reduction in hemicellulose levels in the straw, in turn heightening the compatibility of the lignocellulose material with the PLA and improving the water permeability of the PLA composite samples [23,24]. Solution 2 was then mixed with Solution 1 and gently stirred at 100 °C for 4 h until the solvent evaporated. The consequent product with plastic-like behavior was poured into molds (1.2 cm). The samples were stored at room temperature for 24 h to ensure the gradual evaporation of residual volatile solvents and avoid the rapid onset of shrinkage and fracture during the following curing process. The samples were subsequently dried at 90 °C for 2 h to enhance cross-linking points between the COOH groups of CA and cellulose. Designations of the samples and their composition as dry matter are given in Table 1, while a schematic diagram of the procedure is appended as Supplementary Data S1.

### 2.4. Characterization 

#### 2.4.1. Structural Properties

Analysis of the cross-section morphology of samples, which had been thinly coated with a gold–palladium alloy, was carried out by scanning electron microscopy (SEM) on a Nova NanoSEM 450 unit set at an operating voltage of 10 kV. Changes in the physical structure of selected biocomposites that had been buried in soil were also evaluated. The samples were removed at predetermined intervals from the soil, cleaned and analyzed by SEM. 

Alterations affecting the chemical structure of the biocomposite during degradation in soil were determined by Fourier transform infrared spectroscopy (FTIR) on a Thermo NICOLET 6700 spectrometer, applying the attenuated total reflection (ATR) technique. Equipped with a diamond crystal, the unit was set to a resolution of 2 cm^−1^ and a wavenumber range of 4000–400 cm^−1^. The samples were removed at predetermined intervals from the soil, cleaned and analyzed by FTIR.

#### 2.4.2. Thermal Properties

The thermal stability of the hydrogels was studied by differential scanning calorimetry (DSC) and thermogravimetric analysis (TGA). DSC measurements were recorded under an inert nitrogen flow of 50 mL N2/min^−1^ using a DSC 1 STAR (Mettler Toledo, Belgium) Instrument. Samples of 5 mg were sealed in non-hermetic aluminum crucibles and were tested within the temperature range from −20 °C to 220 °C. The glass transition temperature (T_g_) was determined as the midpoint of the initial change in slope. Melting (T_m_) and cold crystallization (T_cc_) temperatures were denoted as those for the peaks of the corresponding melting endotherm and crystallization exotherm, respectively, as determined during the second heating scan. The degree of crystallinity (X_c_) was calculated according to the following Equation (1):

(1) $$X_{C}\;(\%)=\frac{\Delta Hm-\Delta Hcc}{\Delta Hm0\cdot w}\;\times \;100\;$$

where ∆*Hm* is melting enthalpy, ∆*Hcc* stands for cold crystallization enthalpy, ∆*Hm*0 represents the heat of fusion associated with pure crystalline PLA (93.1 J∙g^−1^) and *w* corresponds to the weight fraction of PLA in the sample [25].

TGA analysis was performed on a Mettler-Toledo TGA/SDTA 851e instrument under nitrogen flow (10 mL/min^−1^). Samples of 5 mg were sealed in platinum crucibles, and a heating rate of 10 °C min^−1^ was applied at intervals, with temperatures ranging between 25 °C and 500 °C.

#### 2.4.3. Swelling Ratio and Water Retention in Soil

Values for the swelling ratio (SR) of the PLA/lignocellulose biocomposite were assessed by the gravimetric method in distilled water. A dried sample was soaked in distilled water for 24 h at room temperature (24 °C). The kinetics of swelling were discerned by taking the soaking material out of the solution at defined intervals of time (1, 30, 60 and 1440 min.), removing excess media with filter paper and recording the weight. Values for SR were determined by weighing the samples before and after immersion in distilled water for 24 h by using the following Equation (2):

(2) $$SR\left(\%\right)=\left(\frac{Ws-Wd}{Wd}\right)\times 100$$

where *Ws* and *Wd* are the masses of swollen and dried samples (g), respectively.

The swelling capacity of the material was also evaluated in soil extracts, with the latter prepared according to the following procedure [26]. In brief, 100 g of air-dried commercial garden soil (pH 5.8–6.0) was dissolved in 500 mL of deionized water in an incubator shaker for 60 min at 165 rpm and centrifuged for 7 min at 1000 rpm. The supernatant was then separated out by filter paper (55 mm) from any remaining soil particles. The supernatant obtained was employed as a swelling medium, and values for SR were calculated according to Equation (2). 

The efficiency of the prepared material for retaining water in soil was gauged as follows: 1 g of sample was thoroughly mixed with 50 g of dry soil and placed in a pre-weighted pot (*W*); soil without any sample present was applied as the control (*W*_0_). In total, 30 mL of distilled water was added afterward, and the weight was recorded again (*Wt*). The extent of water retention in soil (WR, in percent) was calculated by the following Equation (3).

(3) $$\mathrm{WR}\;(\%)=\left(\frac{Wt-W}{W0-W}\right)\times 100$$

The above experiment for SR and WER was repeated to investigate the reusability of the material in the soil.

#### 2.4.4. Degradation Studies

A soil burial test and GPC analysis of degraded samples were employed to characterize the absorbent material in order to study the effect of its composition on degradability in the soil environment. The degradation of the biocomposites in soil was assessed via a burial method under a controlled temperature (25 °C), whereby hydrogels were buried at a depth of 3 cm from the surface in a pot containing soil. Relative humidity was maintained at 40%. The samples were removed from the soil at 5-day intervals, washed gently with water to remove soil particles, cleaned and dried at 45 °C for 48 h in a vacuum oven. The degradation of the samples was calculated by the following Equation (4):

(4) $$D\;(\%)=\frac{Wi-Wf}{Wi}\times 100$$

where *Wi* is the initial weight of samples prior to any degradation, and *Wf* refers to the weight of a sample after specific periods of biodegradation.

The average molecular weight (M_w_) and molecular weight distribution of samples and the changes they underwent during degradation tests were analyzed by gel permeation chromatography (GPC) on an HT-GPC 220 system (Agilent) equipped with a dual detection system (“RI” refractive index and “VIS” viscosity detectors). The samples were dissolved in tetrahydrofuran (THF) (~2 mg.ml^−1^) overnight. Separation and detection took place using a series of mixed columns (1xB, 1xD and 1xE) (300 × 7.8 mm, Polymer Laboratories). Analysis was carried out at 40 °C in THF at a flow rate of 1 mL.min^−1^ and a loading volume of 100 μL. The GPC system was calibrated with narrow polystyrene standards ranging from 580 to 6,000,000 g.mol^−1^ (Polymer Laboratories Ltd., Church Stretton, UK). Values for mean molar mass or molecular weight (M_w_), number of average molar mass (M_n_) and polydispersity index (Ð = M_w_/M_n_) for the tested samples were determined using peaks corresponding to the polymer fraction in accordance with the universal calibration method. All data were processed in Cirrus software (Agilent Technologies, Santa Clara, CA, USA) [27].

### 2.5. Statistical Analysis

All experiments were performed in triplicate at least three times. All data are expressed as the mean (± standard deviation (SD). Standard deviations were calculated for selected parameters according to Equation (5) and represented as error bars.

(5) $$\mathrm{SD}=\sqrt{\frac{\sum_{i=1\;}^{n}\left(x-x_{i}\right)}{n-1}}$$

## 3. Results and Discussion 

### 3.1. Morphological Analysis

**Figure 1:**
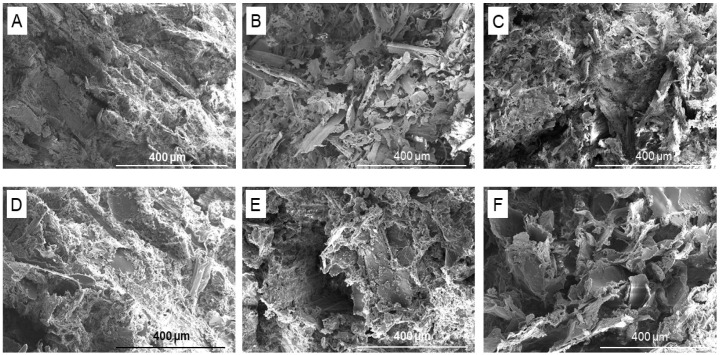
SEM micrographs of the inner structure of samples with different contents of lignocellulose-based fillers; (**A**–**C**) samples containing 15, 35 and 60 wt% WS, respectively; (**D**–**F**) samples with 15, 35 and 60 wt% SD, respectively.

Figure 1 shows that cross-section morphology was largely affected by an increase in the content of the lignocellulose-based filler. Samples with an increased amount of WS or SD possessed numerous voids and cavities, whereas those with 15% WS/SD appeared more homogeneous and compact, as the subsequent PLA that possessed greater solidity and cross-linking in the PLA-lignocellulose material was denser [24]. A simple heat-induced mechanism pertained to such cross-linking (the esterification of cellulose by citric acid), whereby citric acid dehydrated over two reactive anhydride stages, creating cross-links between adjacent cellulose chains [5]. The resultant material exhibited great strength and stability, albeit at the expense of reduced absorption capacity. However, a sample with a high amount of fillers had a porous structure with pore sizes of 100–200 microns, facilitating the absorption and retention of water molecules, which diffused into the framework of the material and heightened the swelling ratio and extent of water retention in the soil environment [28]. 

**Figure 2:**
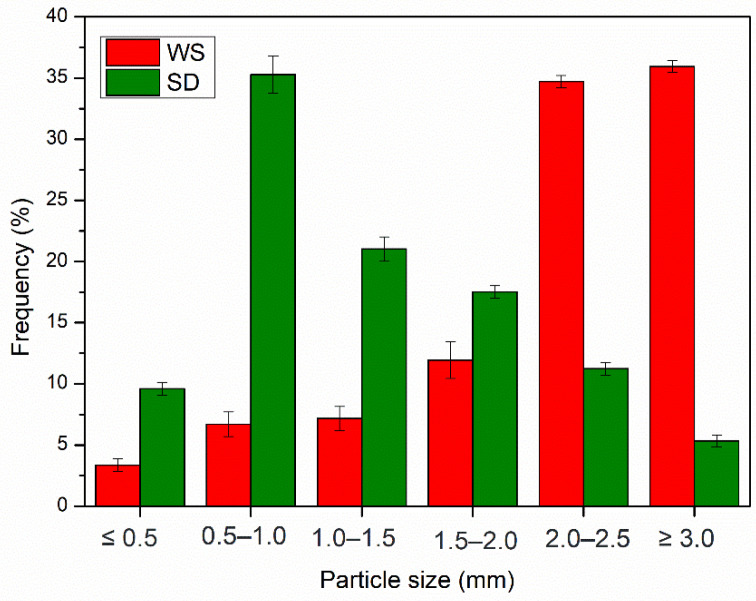
Particle size distribution (in mm) of WS and SD in biocomposites.

Differences in particle size potentially affect the structural properties and subsequent swelling capacity of a material. Particle size fractionation revealed that particles of the ground WS generally measured 2.0–2.5 mm (35%) and 3 mm (36%), while SD accounted for 35% of particles of 0.5–1.0 mm and 21% of those exceeding 1.0–1.5 mm in size as depicted in Figure 2. 

### 3.2. Structural Analysis

**Figure 3:**
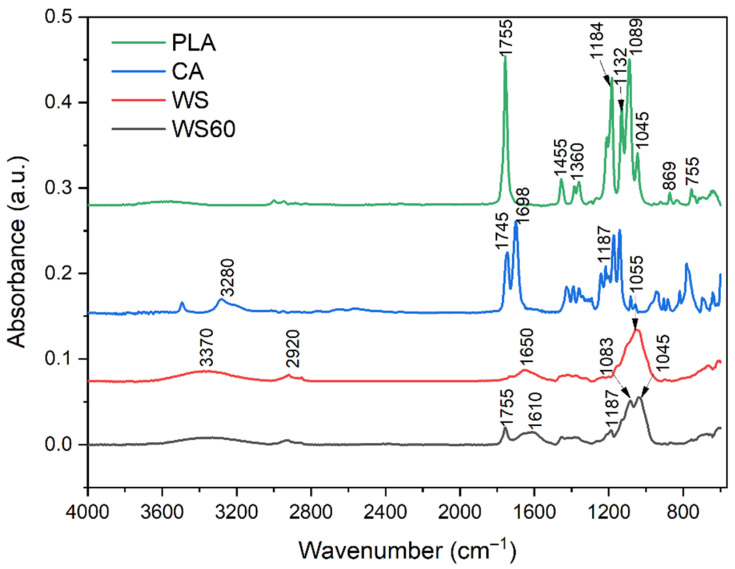
FTIR spectra for the low-molecular-weight PLA, WS, CA and cross-linked sample (WS60).

**Figure 4:**
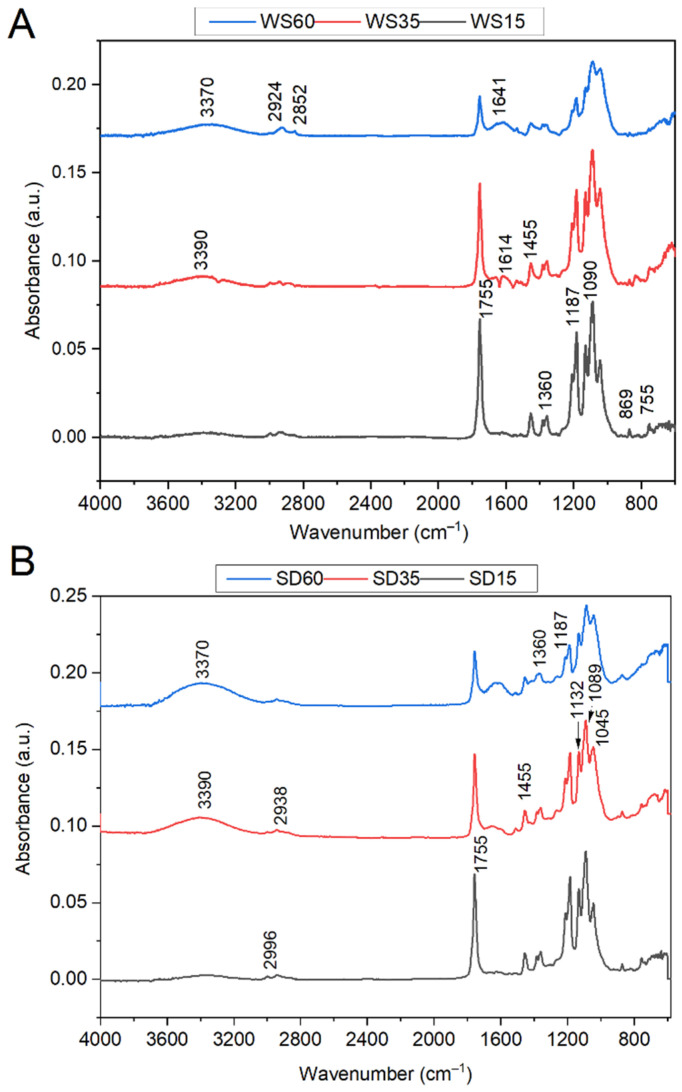
FTIR spectra for samples with different amounts of WS (**A**) and SD (**B**).

The typical FTIR–ATR spectra of the synthesized low-molecular-weight PLA, WS and PLA-WS and PLA-SD samples presented in Figure 3 and Figure 4A,B are in agreement with results reported elsewhere [25,29,30,31]. The positions of major absorption peaks and corresponding functional groups for the straw are as follows: the one at 3370 cm^−1^ pertains to the stretching vibration of intermolecularly associated hydroxyl in the cellulose (-OH); 2920 cm^−1^ is the C-H stretching vibration of the saturated alkyl groups of the cellulose and hemicellulose; the peak at 1651 cm^−1^ correlates with the carbonyl group of the acetyl ester in the hemicellulose and carbonyl aldehyde in lignin [32]. The absorbance peak at 3280 cm^−1^ is assigned to the O–H stretching vibration of the hydroxy group of CA [33]. In Figure 4A,B, a broad peak between 3500 and 3000 cm^−1^ is attributed to the stretching vibration of the -OH groups of CA and lignocelluloses [34]. The absorbance band at 1755 cm^−1^ is attributed to a C=O stretching vibration of PLA [25] and is weaker for samples with a greater content of the lignocellulose filler. The weak band at 1650 cm^−1^ that occurred in WS correlates with the carbonyl group of the acetyl ester in the hemicellulose and carbonyl aldehyde in the lignin [32]. The broad weak band at 1610 cm^−1^ relates to the aromatic skeleton vibrations of lignin characteristics in biomass WS [35]. The peak at 1455 cm^−1^ typically denotes a CH_3_- band, and an asymmetric one appears at 1360 cm^−1^ [25]. The region between 1300 and 1050 cm^−1^ shows four intensive absorption peaks at 1184, 1132, 1089 (C-O-C stretching) and 1045 cm^−1^ (-OH band). The peak at 1055 cm^−1^ (WS sample) denotes the bending vibration of -OH and the stretching vibration of C-O in saccharide cellulose [30]. The peaks located at 869 and 755 cm^−1^ correspond to -C-C- stretching. The intensities of all such peaks diminished in relation to the concentration of PLA in the sample, as presented in Figure 4. 

The absorbance band at 1626 cm^−1^ (Figure 4) indicated absorbed water in the samples. This band shows reduced intensity when citric acid was present, and this is probably caused by the involvement of hydrophilic groups in cross-links with CA; thus, the films absorbed less water [31].

Minor absorption peaks appear between 1500 cm^−1^ and 1400 cm^−1^, reflecting unsaturated (C=C) in the molecules and potentially relating to waxes, extractives and lignin, and they are in agreement with those for lignocellulosic natural fibers. This region can also be characterized by aromatic ring vibrations and ring breathing associated with C-O stretching in the lignin [32].

### 3.3. Thermal Properties

The thermal properties of neat PLA and PLA composites were studied by DSC, and the data collected are summarized in Table 2. The DSC results revealed that increasing the concentration of WS and SD particles in the sample reduced the T_g_ and melting temperature (T_m_) of the matrix; the respective causes were the plasticizing influence of the lignin and the heightened formation of imperfect crystals during the accelerated crystallization process of biocomposites [6]. The crystallization temperature (T_c_) of the biocomposites increased (106 °C ≥ 125 °C) in contrast with pure PLA (T_c_ = 102 °C), revealing that nucleation by lignocellulose fibers hastened the PLA crystallization process [6]. The data given in Table 2 show that the presence of lignocellulose fillers raised the crystalline mass fraction (X_c_) of samples. The greater X_c_ of the PLA-WS and PLA-SD composites evidences the better compatibility of PLA macromolecules with the lignocellulose particles of WS or SD, directly resulting in an increase in accessible heterogeneous nucleation sites [36]. The increased crystallinity observed for all samples could also relate to the lower amount of amorphous lignin and hemicellulose present in untreated lignocellulose fibers [37].

**Figure 5:**
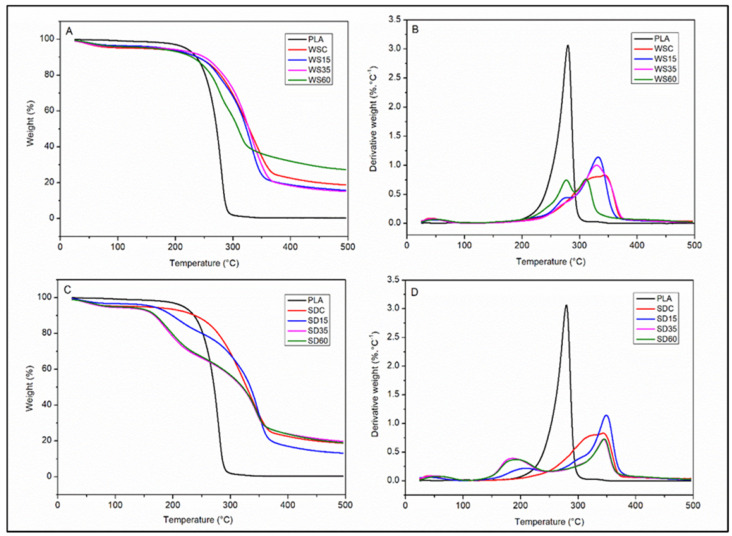
TGA (**A**,**C**) and dTGA (**B**,**D**) thermograms for neat PLA and PLA-WS/PLA-SD composites.

The thermal degradation of samples containing WS and SD at different concentrations were compared with neat PLA. As detailed in Figure 5A,C, the TGA curves for the loss in mass at 500 °C show that both lignocellulose fillers enhanced thermal stability by up to 21% and 28% in WS60 and SD60, respectively, compared to pure PLA; the likely causes are the strengthening properties of lignocellulose fibers and the cross-linking reaction of CA with the carboxylic groups of lignocellulosic materials, with the possible outcome of protecting WS60 composites against degradation by temperature. The highest thermal effect was observed for samples WS15 and SD15. Those supplemented with 15% of the lignocellulose-based fillers were stable without a significant loss in mass at up to 349 °C. In parallel with the increase in the amount of filler, a slight drop-off was observed in the maximum temperature for degradation. Findings showed that the primary effect of increasing the content of lignin reduced the degradation of PLA-WS and PLA-SD samples. The thermal stability of non-cross-linked WSC and WSD samples was slightly less than cross-linked composites, highlighting the influence of cross-linking on thermal degradation. 

Compared to pure PLA, the T_max_ of the biocomposite increased by up to 53 °C (15 wt% sample); however, increasing the content of the fillers led to a small reduction in T_max_, and this was probably due to the greater presence of less thermal stable components in WS or SD [37]. Taking samples with 60 wt% of filler as an example, the addition of WS reduced the maximum degradation temperature by up to 21 °C, which is over the degradation temperature observed with 15 wt% of the supplemented lignocellulose. Other authors have also reported a reduction in the thermal stability of biopolyesters by introducing cellulosic fibers [37,38]. The thermal degradation values are summarized in Table 3. 

The TGA thermograms in Figure 5A,C plot the rate of change in mass and temperatures with the maximal loss in mass (T_max_). The dTGA curves in Figure 5B,D depict the degradation process in three stages: the first stage (below 100 °C) primarily involves the removal of the absorbed moisture, the second stage (150–300 °C) relates to the degradation of cellulosic substances such as hemicellulose and cellulose and the final stage (310–400 °C) pertains to the degradation of non-cellulosic materials in fibers [36]. The findings demonstrate that the temperature for decomposition by biocomposites is higher than that for PLA, with a trend for a gradual decrease in temperature that is in parallel with the increase in WS and SD content.

### 3.4. Swelling Properties

**Figure 6:**
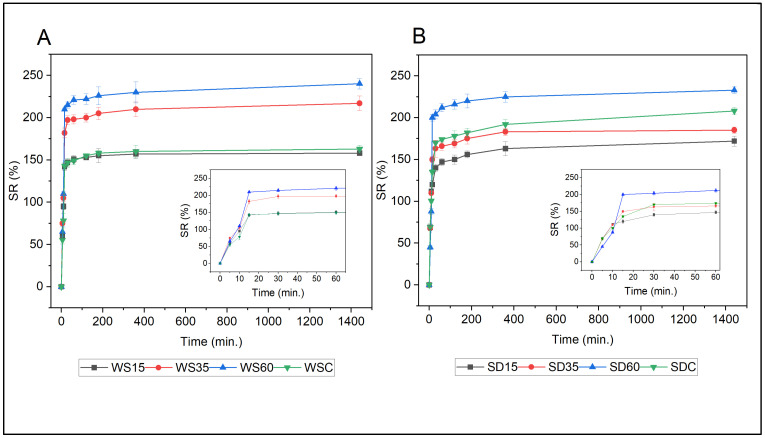
Swelling ratios (SR in percent) of PLA-WS (**A**) and PLA-SD (**B**) composites over a period of 24 h of immersion in distilled water. The inserted graphs detail the trends for SR in the first 60 min of swelling.

**Figure 7:**
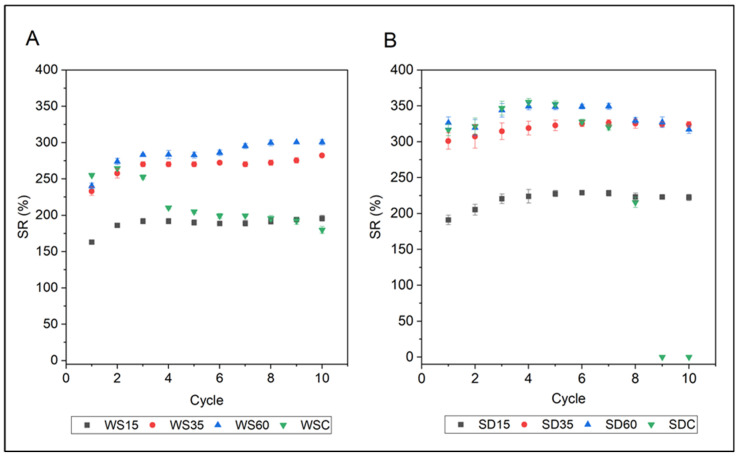
Re-swelling ability of PLA-WS (**A**) and PLA-SD (**B**) samples in DW during 10 cycles of swelling/drying.

The water absorption characteristics of the prepared biocomposites with different contents of lignocellulose fibers are given in Figure 6 and Figure 7. The graphs show that the swelling ratios (SR) of biocomposites increase in line with the increase in WS or SD concentrations. Variances in the swelling kinetics of PLA-SW and PLA-SD materials are visible in Figure 6, and they are probably initiated by the interactions of hydrophilic lignocellulose fibers with the PLA matrix [39]. With the increase in the content of WS or SD in the composite, free -OH groups present in the cellulose and hemicellulose structure absorb water through hydrogen bonds that form between the water molecules and OH-groups on the surfaces of WS or SD particles [11]. Heating the lignocellulose biomass when preparing the biocomposites led to an increase in the porosity of the surface structure of the biomass through a partial degradation of hemicellulose and lignin, facilitating the penetration of composite materials by water molecules [24,40]. This resulted in high water absorption in samples with higher concentrations of WS or SD. As Figure 6 shows, a rapid increase in water absorption occurred during the initial stage of swelling, followed by a gradual course of absorption over 24 h until the samples reached an equilibrium. The highest SR was observed for samples WS60 (SR 240%) and SD60 (SR 233%).

The data presented in Figure 7 reveal that all samples were able to absorb and desorb water repeatedly, indicating their efficiency for water retention and reusability. The SR of PLA-WS/SD composites was evaluated during 10 cycles of swelling and drying. A lower capacity for water absorption was observed for samples supplemented with fewer lignocellulose fillers, with a slight increase in SR from 163 to 195% for WS15 and from 191 to 222% for SD15 in comparison to biocomposites with higher concentrations of fillers. This significant reduction in the ability to absorb water was caused by the hydrophobic nature of PLA. Samples with greater concentrations of lignocellulose fillers exhibited a slightly heightened capacity for water absorption, with WS35 and WS60 reaching SR values of 282% and 301% in the 10th cycle of swelling and drying, respectively. A similar trend was observed for PLA-SD composites, where SR increased with each swelling and drying cycle. The SR of SD35 slightly increased from 301% to 324% compared to the SR of SD60, which slightly decreased from 326% to 316% in the last such cycle. As observed in Figure 7, however, the SR of these PLA composites cross-linked with CA remained almost constant throughout the 10 cycles of swelling and drying, indicating good stability and reusability in soil. In contrast, a significant reduction in absorption capacity was observed for the non-cross-linked WSC (255 > 179%) and SDC samples, with the latter starting to disintegrate after the fifth cycle, probably due to its less stable structure and the breakup of particles during swelling tests. The samples blended with 35% and 60% of WS or SD showed far higher water absorbency as the cycles continued. 

### 3.5. Water Retention in Soil

**Figure 8:**
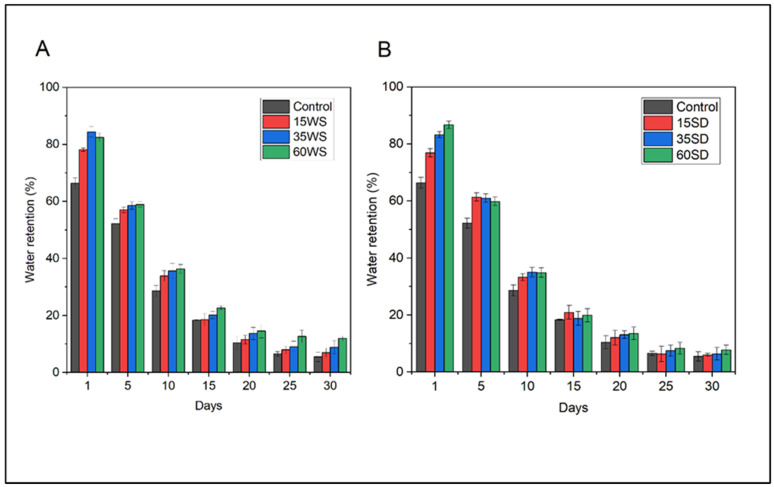
Water retention capacity of soil (%) after the application of PLA-WS (**A**) and PLA-SD (**B**) composites. Soil absent of any such additive constituted the control sample.

The results presented in Figure 8 show that adding PLA-WS/SD composite samples to the soil improved its water-holding capacity and slowed down the evaporation of irrigation water from the soil during drought conditions for at least 30 days. The soil containing PLA composites with lignocellulose fillers held and retained more water than pure soil (the control); soil samples designated as WS60 and SD60 retained over 85% of irrigated water compared to samples with low lignocellulosic content (WS15 and SD15), which demonstrated 72% and 74% in this regard at the beginning of the experiment. The water retention capacity of the control soil just equaled 66% at the initial phase of swelling, with a decreasing trend during 30 days. A slight increase in water retention capacity was observed in soil samples containing the WS absorbent material (12%) in comparison with the PLA-SD (8%) and control soil samples (5.5%) at the close of the experiment.

### 3.6. Biodegradability

**Figure 9:**
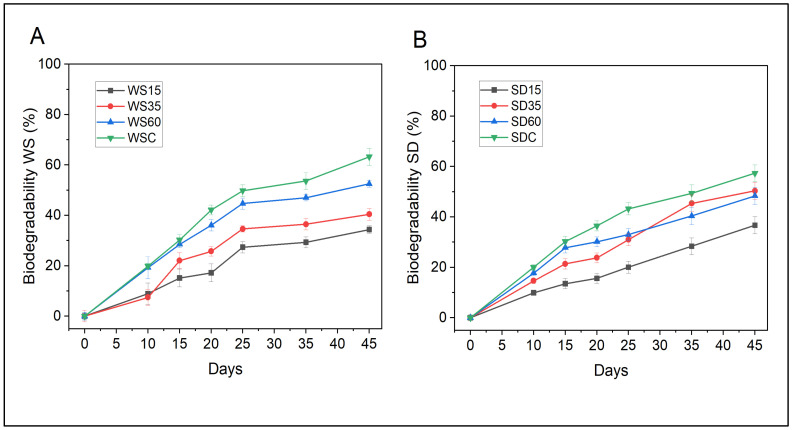
Biodegradability (in percent) of the PLA-WS (**A**) and PLA-SD (**B**) composites during soil burial tests. The plots relate to a loss in the mass of PLA-WS/SD within 45 days.

Although PLA has been verified as a naturally degradable plastic in soil or compost, it has been shown to be less susceptible to microbial attacks and degradation in a natural environment than compared to other biodegradable biopolymers [6]. The biodegradability of PLA-WS and PLA-SD composites in soil was investigated using a soil burial test, and the results are presented in Figure 9. 

The biodegradation of PLA-WS and PLA-SD composites took place over three distinct phases: water evaporation; the breakdown of side chains in the PLA/lignocellulosic network; and the decomposition of main chains, including cellulose and PLA [41]. When PLA/lignocellulose-based composites were buried in soil, it was observed that the cross-linking structure of the polymer network broke down, and small fragments were obtained after 45 days of the experiment. Changes in the visual appearance of the biocomposites during the soil burial test are depicted in Figure S2 in the Supplementary Materials.

The plots in Figure 9 show that the presence of the low-molecular-weight PLA slowed down the rate of the biodegradation of biocomposites, and this is probably because PLA is hydrophobic in nature, which makes it less susceptible to microbial attacks and degradation in a natural environment. The slow biodegradation process is also explained by the glass transition temperature (*T*_g_) of PLA, as it exceeds ambient temperatures, and microorganisms capable of degrading PLA are scarce in the given environment [6]. The course record for sample WS15 was one of extended biodegradation, reaching almost 34% after 45 days in soil. The sample with the same content of SD was similar, achieving 37% of degradation by the close of a 45-day period. In contrast, samples with 35% and 60% of the same filler showed a trend of increase, and this was most likely caused by the hydrophilic lignocellulose that absorbed more water, resulting in high water evaporation and faster degradation. 

A study in the literature describes the environmental degradation of paddy straw/PLA biocomposites, wherein a similar, considerable biodeterioration of the studied materials was observed, particularly those containing higher loadings of lignocellulosic fillers; the phenomenon was attributed to the hydrophilic nature of the straw, leading to high water uptake and relatively stronger colonization and activities of microorganisms [42]. Indeed, a PLA and wood powder/starch composite exhibited a rapid rate of degradation as the starch ratio went up, leading to effective degradation within only 75 days [42]. Introducing natural components such as fillers, therefore, could promote the biodegradability of PLA composites. It should be taken into account, though, that a variety of factors might influence the degradation of composites, e.g., particle size, the geometry of the sample, the degree of crystallinity and molecular weight, as well as environmental conditions such as temperature, pH and microbial activity [43].

Herein, samples WS60 and SD60 showed 52% and 50% degradation after 45 days of the study, respectively, within which time 63% WSC and 57% SDC had degraded. These results emphasize the more rapid biodegradability of the latter non-cross-linked materials with high lignocellulose content and make it evident that cross-linking with CA directly affects the biodegradation process.

**Figure 10:**
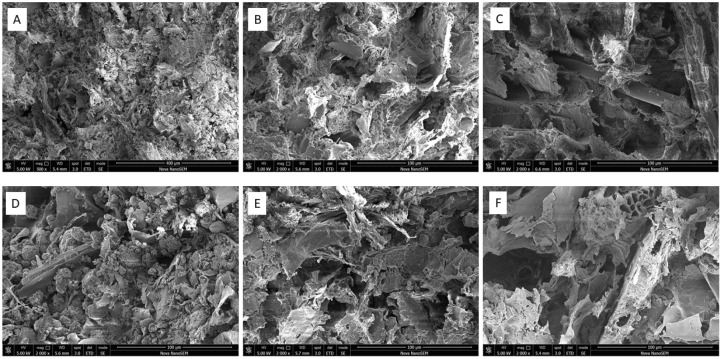
Structural changes to the inner structures of PLA-WS and PLA-SD composites after degradation, as discerned by SEM; (**A**–**C**) refer to sample WS35 and (**D**–**F**) to SD35; (**A**,**D**)—neat sample; (**B**,**E**)—sample after 20 days in soil; (**C**,**F**)—sample after 40 days in soil. The scale bar equals 100 µm.

The SEM micrographs of the PLA-WS/SD composites in Figure 10 show the formation of cavities in the inner structure during the degradation process in soil. As visible in Figure 10B,C,E,F, the number of cavities in the materials increased over time, causing the disintegration of the samples during their degradation in the soil, with the probable causes being environmental conditions such as humidity, temperature and soil-related microbial activity [43].

**Figure 11:**
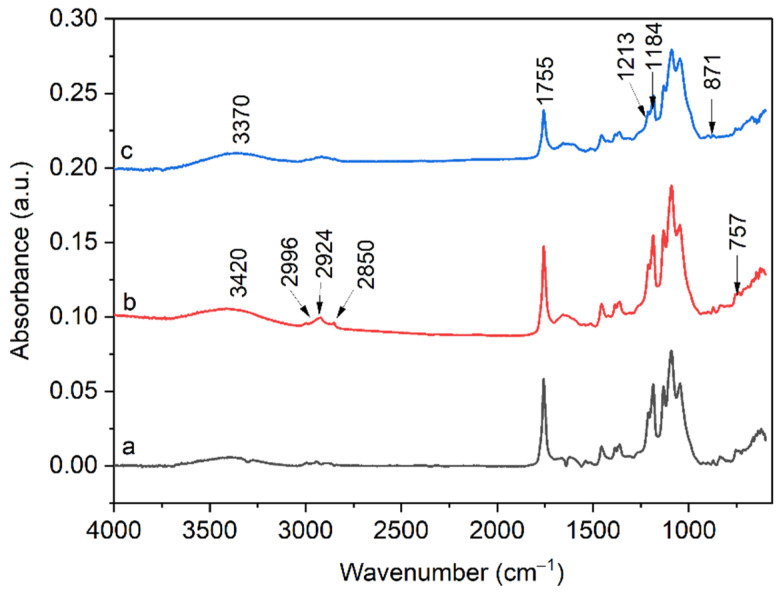
FTIR spectra for PLA-WS composite WS35 (**a**) after 35 (**b**) and 50 days (**c**) in soil, respectively.

Alterations in the chemical structures of PLA-WS composites during biodegradation were also confirmed by FTIR analysis. Figure 11 shows the FTIR spectra for the WS35 sample after 35 and 50 days of biodegradation in soil. The FTIR spectra for degraded samples show peaks at 3420, 3370, 2996, and 2924, which potentially indicate the formation of the free hydroxyl groups of CA and lignocellulose during the microbial attack of samples in soil. The intensity of some peaks for degraded PLA-WS composites was lower than those described above in Figure 4. For example, the region of 1755 cm^−1^ represents an expression of ester groups formed by the occurrence of cross-linking with citric acid. As the samples degrade, the strength of their carboxyl bonds diminishes. A new wide absorption band occurs at 1639 cm^−1^ for the degraded samples, attributed to the water absorbed by cellulose during degradation [29]. The characteristic peaks of C–O stretching at 1213 cm^−1^ and 1184 cm^−1^ were also reduced, suggesting that disintegration takes place with respect to the aforementioned ester bonds in the cross-linked sample [44]. The peaks located at 871 and 750 cm^−1^ correspond to -C-C- stretching, while the peak at 871 cm^−1^ constitutes the amorphous phase, and the peak at 750 cm^−1^ constitutes the crystalline phase [25]. After 50 days of degradation, the peaks almost disappeared. 

The degradation of samples in terms of change in molecular weight was gauged by GPC, as detailed in Table 4. Analyses took place at certain time intervals during degradation tests in soil. Molecular mass differed according to the content of lignocellulose, and biocomposites with 15% of WS/SD demonstrated the lowest values at the end of the experiment. Samples WS15 and SD15 degraded by approximately 50% after 50 days in soil. Within the same time frame, the molecular weight of cross-linked WS60 and SD60 samples dropped by up to 45–47%, compared to WSC (51%) and SDC (56%), evidencing the influence of cross-linking on the decomposition of materials in the soil. Another potential explanation was the presence of lignocellulosic materials due to cellulose possessing a high degree of crystallinity that hindered enzymatic actions, in addition to the association of cellulose and hemicellulose with lignin, whereby a natural barrier was created for the cellulosic portion against the attack of microorganisms [30]. It is evident from these findings that GPC analyses provided greater objectivity for assessing the degradation of biocomposites than gauging a loss in mass by the burial test. The molecular weight of neat PLA reduced from 7700 g/mol^−1^ to 6300 g/mol^−1^ after being buried in the soil for 50 days. The reason was probably the active microorganisms and moisture in the earth that could easily penetrate PLA molecules; this led to the disruption of the molecular architecture promoted by the random hydrolytic scission of ester bonds in the amorphous chains of the PLA during the degradation process [43]. Such degradations also caused an increase in the PDI of neat PLA from 1.6 to 2.4 and an increase from 1.5 to 4.0 for biocomposites. 

## 4. Conclusions

PLA biocomposites supplemented with different lignocellulose-based filler content were prepared herein, employing widely available, low-cost materials and a simple fabrication process and without any pre-treatments involving the extraction of raw cellulosic materials. The results show that combining raw hydrophilic lignocellulose biomass with PLA promotes the swelling capacity of the given biocomposites. Samples with the greatest amount of WS or SD exhibited a swelling ratio of up to 300% along with a capability for re-swelling, indicating the reusability of the materials and their potential for use in agriculture as a soil conditioner. Placing such absorbent materials in the soil allowed their retention of humidity under drought conditions for periods that were almost 10 times longer than the control, i.e., soil without the material that had merely been watered. The results of this work clearly demonstrate that PLA biocomposites supplemented with a filler of lignocellulose biomass degrade easily. Samples with 60% of WS or SD had broken down by 63% and 57%, respectively, by the close of a 50-day period. Employing such environmentally friendly PLA-lignocellulose biocomposites in agriculture could greatly benefit the soil environment and the crops grown in it as a consequence of the swelling properties of such organic, readily biodegradable materials.

## Figures and Tables

**Table 1:** Composition of samples.

	Sample Designation	PLA Solution (% *w*/*v*)	Cross-Linking Agent (%) *	Residual Biomass (%) **
	PLA ***	-	-	-
Wheat straw (WS)	WS15	10	10	15
	WS35		10	35
	WS60		10	60
	WSC		0	60
Sawdust (SD)	SD15		10	15
	SD35		10	35
	SD60		10	60
	SDC		0	60

* wt% related to the amount of PLA. ** wt% related to the amount of PLA solution. *** Pure PLA fabricated by a procedure described in the “PLA synthesis” section.

**Table 2:** Results of DSC thermograms for PLA and the PLA composites with different amounts of WS and SD.

Formulation	*T*_m_ (°C)	*T*_g_ (°C)	*T*_c_ (°C)	*X*_c_ (%)
PLA	135	50	102	2
WS15	145	53	106	5
WS35	148	55	117	13
WS60	149	55	121	20
WSC	143	52	120	21
SD15	138	47	117	13
SD35	142	49	123	20
SD60	143	49	125	22
SDC	135	48	119	24

**Table 3:** TGA data for the PLA-WS/SD composites.

Formulation	T_max_ (°C)	Total Loss in Mass at 500 °C (%)
PLA	279	99
WS15	332	84
WS35	329	84
WS60	311	72
WSC	326	82
SD15	349	86
SD35	344	79
SD60	345	80
SDC	341	81

**Table 4:** Molecular weights and polydispersity indexes of neat PLA and biocomposites during degradation tests under aerobic conditions in the soil.

Days	0		15		30		45	
Sample	Mw (g.mol^−1^)	Mw/Mn (Ð)	Mw (g.mol^−1^)	Mw/Mn (Ð)	Mw (g.mol^−1^)	Mw/Mn (Ð)	Mw (g.mol^−1^)	Mw/Mn (Ð)
PLA	7700	1.64	7000	1.84	6500	2.23	6300	2.41
WS15	10,000	1.59	9400	1.57	8000	2.36	5000	3.30
WS35	9900	1.53	8300	1.44	7300	3.04	5200	3.67
WS60	10,600	1.59	8200	1.38	6600	3.74	5100	4.10
WSC	10,900	1.55	8200	1.75	6300	2.87	5600	3.99
SD15	9900	1.59	8400	1.57	7000	2.90	5200	3.61
SD35	9700	1.57	8900	1.50	7500	3.22	6000	3.48
SD60	10,900	1.51	9000	1.36	8300	3.36	5100	3.87
SDC	10,800	1.55	9700	1.45	7900	3.14	6000	3.94

## Data Availability

The data presented in this study are available in the article.

## Supplementary Materials

The following supporting information can be downloaded at: https://www.mdpi.com/article/10.3390/polym15102243/s1. Figure S1: Schematic procedure of the sample preparation; (1a) wood sawdust, (1b) milled wheat straw, (2) dissolution of PLA in acetone to obtain 10% *w*/*v* solution, (3) addition of the relevant amount of residual biomass into PLA/citric acid solution, (4) dried PLA/WS(SD) samples after curing at 90 °C. Figure S2: Visual changes of biodegradation of the PLA-WS biocomposites in soil: (A) WS15, (B) WS35, (C) WS60, during 10, 20 and 40 days, resp.
