# Supplementary material for: Renewable Poly(Lactic Acid)Lignocellulose Biocomposites for the Enhancement of the Water Retention Capacity of the Soil
Source: Polymers (Basel). 2023 May 9;15(10):2243. doi: 10.3390/polym15102243 (PMC10220557; doi:10.3390/polym15102243)
Supplement: Supplementary file 1 [file polymers-15-02243-s001.zip › polymers-2349148-supplementary.pdf]

Supplementary materials

Renewable Poly(Lactic Acid)Lignocellulose Biocomposites for the Enhancement of the Water Retention Capacity of the Soil

Dalila Rubicela Cruz Fabian [1], Silvie Durpekova [1,*], Miroslava Dusankova [1], Jaroslav Cisar [1], Petra Drohsler [1], Ondrej Elich [2], Marketa Borkova [2], Jarmila Cechmankova [3] and Vladimir Sedlarik [1]

1 Centre of Polymer Systems, University Institute, Tomas Bata University in Zlin, Tr. T. Bati 5678,
760 01 Zlin, Czech Republic; cruz_fabian@utb.cz (D.R.C.F.); dusankova@utb.cz (M.D.); jcisar@utb.cz (J.C.); pvalkova@utb.cz (P.D.); sedlarik@utb.cz (V.S.)

2 Dairy Research Institute, Ke Dvoru 12a, 160 00 Prague, Czech Republic; elich@vumlekarensky.cz (O.E.); borkova@milcom-as.cz (M.B.)

3 Research Institute for Soil and Water Conservation, Zabovreska 250, 15627 Prague, Czech Republic;
cehmankova.jarmila@vumop.cz

* Correspondence: durpekova@utb.cz

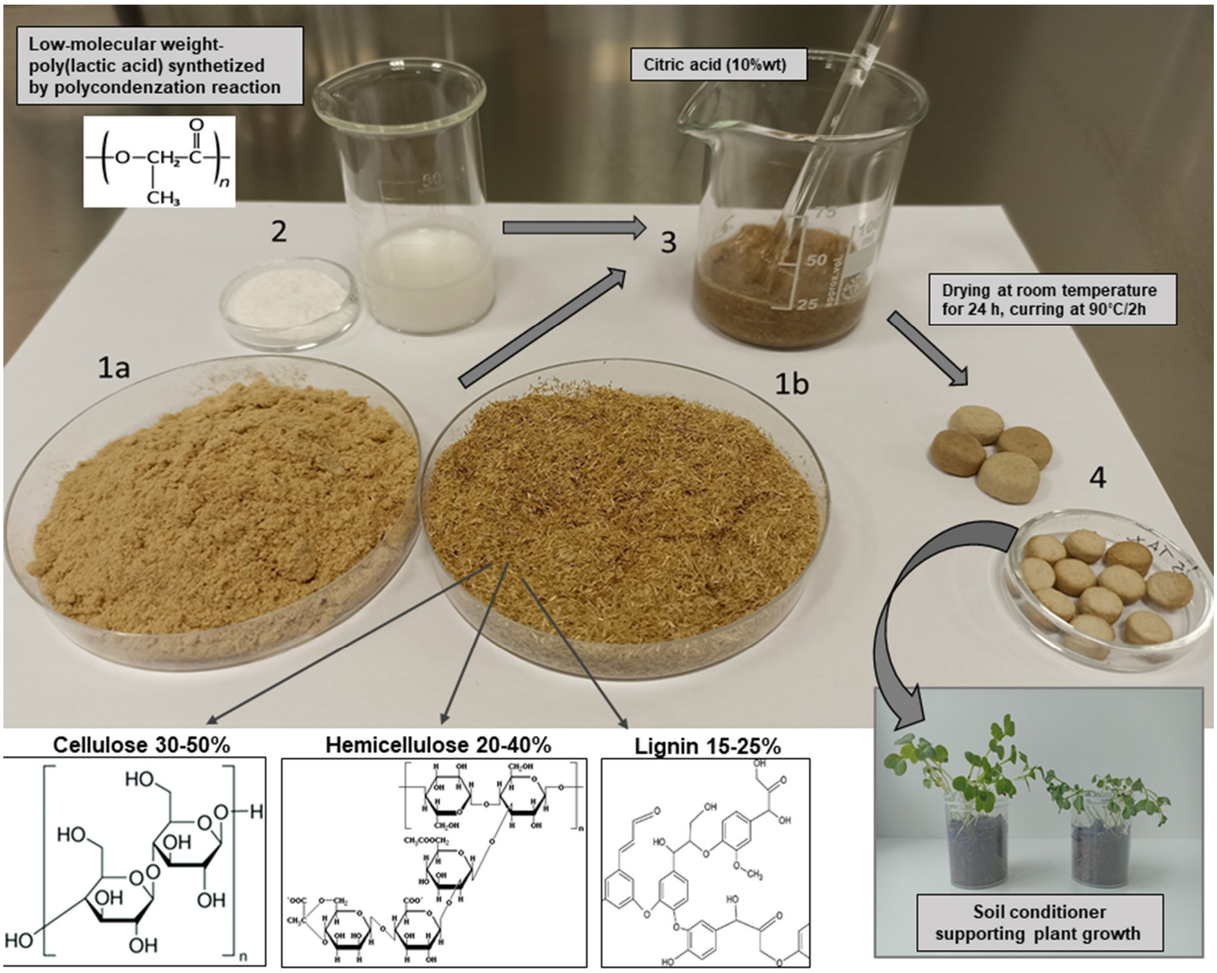

Figure S1. Schematic procedure of the sample preparation; 1a) wood sawdust, 1b) milled wheat straw, 2) dissolution of PLA in acetone to obtain 10% w/v solution, 3) addition of the relevant amount of residual biomass into PLA/citric acid solution, 4) dried PLA/WS(SD) samples after curing at 90°C.

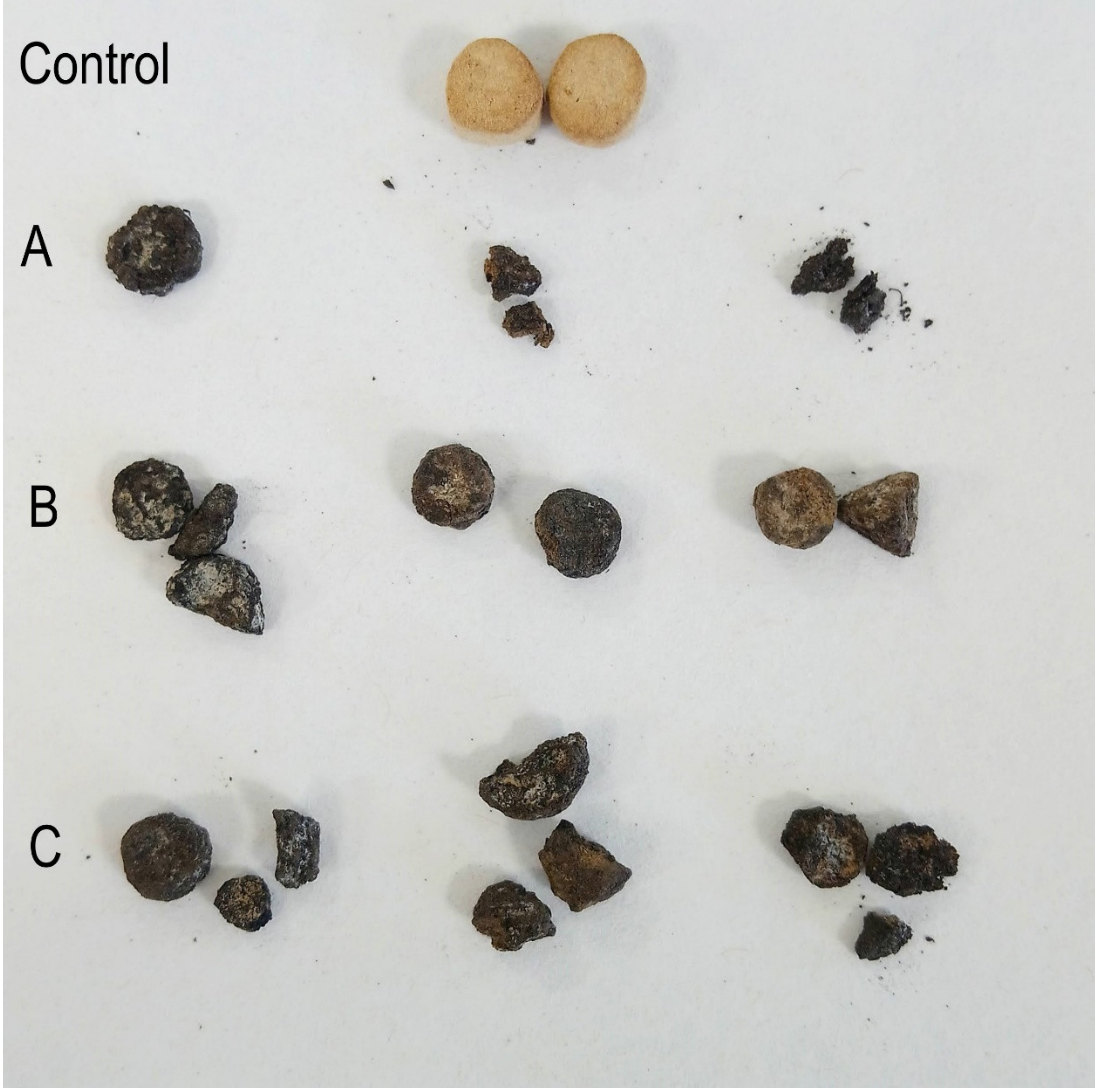

Figure S2. Visual changes of biodegradation of the PLA-WS biocomposites in soil: (**A**) WS15, (**B**) WS35, (**C**) WS60, during 10, 20 and 40 days, resp.
